# Some Practical Suggestions

**Published:** 1907-06-15

**Authors:** E. C. Sherman


### Some Practical Suggestions.

BY E. C. SHERMAN, D. D. S.

*Sea Weed Tents.*—This substance can be had of the druggist, and is tough and hard like a piece of rawhide. Cut it into wedges and use to separate the teeth for inlay work. It may be left safely for from 12 to 48 hours. It is especially good where the teeth have approximated each other because of cavities which did not keep the teeth in their normal positions. The tents will swell laterly, or across the grain, two and one-half times their width or thickness. If the cavities are sensitive fill them part full with cement, so as to prevent pressure on the sensitive dentine and pain. The tents may be whittled to any desired form or size. There is no pain from such separation, as the work is done so gradually as to produce no shock.

To make bridge attachments to the cuspid teeth: destroy the pulp, and grind the mesial, lingual and distal surfaces so as to make room for the gold backing. Fit the pin into root canal and solder it to the pure gold backing, which has previously been swedged to form and fit. Then replace on the tooth in the mouth, and carefully burnish to an accurate fit. On the lingual surface of the backing lay pieces of clasp gold, and flow solder to unite them to the backing to form a smooth contour. They will in this way spring to place and hold securely as bases for the dummy teeth.

Use of cement for swedging gold inlay matrices: Secure impression of cavity by using dentalac or modeling compound. Fill this with a stiff cement and let it harden. Insert the cement model thus made in a suitable hand sweger and stamp and swage pure gold plate about 36 gauge with soft unvulcanized rubber. Transfer to tooth, and re-burnish if it should not fit accurately. It is then ready to be filled with gold solder to proper contour. If platinum matrix has been used pure gold solder may be employed. If 22 karat gold matrixes is used, a solder that will flow into it without melting the matrix can be used.

